# miR-10b exerts oncogenic activity in human hepatocellular carcinoma cells by targeting expression of CUB and sushi multiple domains 1 (CSMD1)

**DOI:** 10.1186/s12885-016-2801-4

**Published:** 2016-10-18

**Authors:** Qiao Zhu, Li Gong, Jun Wang, Qian Tu, Li Yao, Jia-Rui Zhang, Xiu-Juan Han, Shao-Jun Zhu, Shu-Mei Wang, Yan-Hong Li, Wei Zhang

**Affiliations:** 1 The Helmholtz Sino-German Laboratory for Cancer Research, Department of Pathology, Tangdu Hospital, The Fourth Military Medical University, Xi’an, 710038 China; 2 Department of Gynecology and Obstetrics, Tangdu Hospital, The Fourth Military Medical University, Xi’an, 710038 China; 3 Department of Pathology, Tangdu Hospital, The Fourth Military Medical University, Xi’an, 710038 China

**Keywords:** Hepatocellular carcinoma, miR-10b, CSMD1, Oncogene

## Abstract

**Background:**

Hepatocellular carcinoma (HCC) is a lethal disease, while the precise underlying molecular mechanisms of HCC pathogenesis remain to be defined. MicroRNA (miRNA), a class of non-coding small RNAs, can post-transcriptionally regulate gene expression. Altered miRNA expression has been reported in HCCs. This study assessed expression and the oncogenic activity of miRNA-10b (miR-10b) in HCC.

**Methods:**

Forty-five paired human HCC and adjacent non-tumor tissues were collected for qRT-PCR and immunohistochemistry analysis of miR-10b and CUB and Sushi multiple domains 1 (CSMD1), respectively. We analyzed the clinicopathological data from these patients to further determine if there was an association between miR-10b and CSMD1. HCC cell lines were used to assess the effects of miR-10b mimics or inhibitors on cell viability, migration, invasion, cell cycle distribution, and colony formation. Luciferase assay was used to assess miR-10b binding to the 3’-untranslated region (3’-UTR) of CSMD1.

**Results:**

miR-10b was highly expressed in HCC tissues compared to normal tissues. In vitro, overexpression of miR-10b enhanced HCC cell viability, migration, and invasion; whereas, downregulation of miR-10b expression suppressed these properties in HCC cells. Injection of miR-10b mimics into tumor cell xenografts also promoted xenograft growth in nude mice. Bioinformatics and luciferase reporter assay demonstrated that CSMD1 was the target gene of miR-10b. Immunocytochemical, immunohistochemical, and qRT-PCR data indicated that miR-10b decreased CSMD1 expression in HCC cells.

**Conclusions:**

We showed that miR-10b is overexpressed in HCC tissues and miR-10b mimics promoted HCC cell viability and invasion via targeting CSMD1 expression. Our findings suggest that miR-10b acts as an oncogene by targeting the tumor suppressor gene, CSMD1, in HCC.

## Background

Hepatocellular carcinoma (HCC) is a significant health problem, contributing to more than 600,000 cancer-related deaths globally each year. Of note, approximately half of these cases occur in China [1]. The major risk factors for HCC are hepatitis B or C virus (HBV and HCV) infection and consumption of alcohol or aflatoxin B1-contaminated food products [2]. Because HCC patients are typcially only diagnosed at advanced stages, surgical therapies are often not an option. Moreover, chemotherapy and radiotherapy are generally ineffective for these patients [3]. However, sorafenib, a multiple tyrosine kinase inhibitor, is the only drug that has demonstrated survival benefits in patients with advanced HCC [4]. While liver transplantation could help HCC patients live longer, the availability of organ donors is a limiting factor. Thus, there is an urgent need to better understand the biology and pathogenesis of HCC for developing novel strategies that allow effective control, prevention, or prediction of treatment responses of this deadly disease.

MicroRNAs (miRNAs) are a class of small endogenous non-coding RNAs that play roles in regulating cell growth, differentiation, embryonic development, and disease progression. At the molecular level, miRNA can directly bind to the 3’-untranslated region (3’-UTR) of their target mRNAs to degrade them and/or repress their translation [5, 6]. Accumulating evidence indicates that miRNA expression is remarkably dysregulated in different human cancers and that miRNAs can act as oncogenes or tumor suppressor genes to regulate tumorigenesis, progression, and metastasis [7–9]. Among them, miRNA-10b (miR-10b) has been reported to be highly expressed in many types of human cancers, such as breast, pancreatic, and nasopharyngeal cancers, malignant glioma, and HCC [10–14]. A previous study using Agilent human miRNA microarray detected significant upregulation of miR-10b in HCC tissues [15]. In this study, we assessed miR-10b expression in HCC and then investigated its oncogenic activity and the underlying molecular mechanisms in HCC cells.

## Methods

### Tissue samples and cell lines

A total of 45 paired human HCC and adjacent non-tumor liver tissues (37 males and 8 females with an average age of 51 years; 40 were HBsAg (+), 24 AFP > 400 ng/mL, 31 had a tumor size ≥ 5 cm) were collected from Tangdu Hospital, The Fourth Military Medical University (Xi’an, China). Surgically resected tissue samples, including HCC with or without liver cirrhosis, were fixed in 10 % buffered formalin and embedded in paraffin. Each case was examined and diagnosed by three pathologists according to the morphological criteria of liver cirrhosis and HCC. HCC samples were graded according to Edmondson’s criteria. None of the patients had received any other therapies such as chemoembolization or chemotherapy before surgery. In this study, we obtained paraffin blocks from each patient and isolated total cellular RNA for detection of miR-10b expression.

The human HCC cell line, HepG2, and a normal human hepatocyte line, HL-7702, were cultured in Dulbecco’s modified Eagle’s medium (DMEM, HyClone, Logan, UT, USA) or RPMI 1640 medium (HyClone) containing 10 % fetal bovine serum (Invitrogen, Carlsbad, CA, USA) at 37 °C in a humidified chamber supplemented with 5 % CO_2_.

### Quantitative RT-PCR

Total cellular RNA was isolated from tissues and cells using a miREeasy FFPE kit (Qiagen, Hilden, Germany) and the RNAsimple Total RNA Kit (Tiangen, Beijing, China) according to the manufacturers’ protocols. Next, these RNA samples were amplified using an ABI 7500 fast Real-Time PCR system (ABI, Foster city, CA, USA) and U6 RNA was used as an internal control for miR-10b expression. The PCR amplification conditions were 95 °C for 15 min and then 40 cycles of 94 °C for 15 s, 60 °C for 30 s, and 70 °C for 35 s. For detection of CSMD1 mRNA levels, aliquots of 1 μg of total RNA samples were reversely transcribed into cDNA using the miScriptII RT Kit from Qiagen and subjected to qPCR amplification of CSMD1 mRNA. CSMD1 primers used were 5’-TCCAGTCATTACCACGGCAC-3’ and 5’-CATGCCCAGCATAGCCATTC-3’. GAPDH was used as an internal control, using primers 5’-GCACCGTCAAGGCTGAGAAC-3’ and 5’-TGGTGAAGACGCCAGTGGA-3’.

### Plasmid construction and luciferase reporter assay

The pmiR-RB-REPORT™ luciferase vector (Riobobio, Guangzhou, China) was used to construct the pMIR-CSMD1 or pMIR-CSMD1-mut vectors. HepG2 cells were cultured in 24-well plates and transiently transfected with 100 ng pMIR-CSMD1 or pMIR-CSMD1-mut vector and 50 pmol hsa-miR-10b mimics (10b-m) or mimics negative control (mnc) using Lipofectamine 2000 (Invitrogen). 10b-m and mnc were purchased from GenePharma (Shanghai, China). Luciferase activity was measured after 48 h using the Dual Luciferase Reporter Assay kit (Promega, Madison, WI, USA) according to the manufacturer’s instructions.

### Gene transfection

10b-m, mnc, Hsa-miR-10b inhibitors (10b-i), inhibitors negative control (inc) as well as CSMD1 siRNA were obtained from GenePharma (Shanghai, China). HepG2 cells were cultured in antibiotic-free medium and grown overnight and then transfected with 10b-m, mnc, 10b-i, inc, or CSMD1 siRNA using Lipofectamine 2000 (Invitrogen, Carlsbad, CA, USA) according to the manufacturer’s instructions. The CSMD1 siRNA sequences were 5’-CCAUAUGGCUAACUGGCAUTT -3’ and 5’-AUGCCAGUUAGCCAUAUGGTT -3’.

### Cell viability assay

Cell viability was assessed using the 3-(4,5-dimethylthi azol-2-yl)-2,5-diphenyltetrazolium bromide (MTT) assay. Briefly, cells were seeded into 96-well plates and grown overnight, and then transfected with 10b-m or negative control (NC) using Lipofectamine 2000 (Invitrogen) for up to 4 days. At the end of each experiment, 20 μl MTT was added into each well and cells were incubated for 4 h at 37 °C. Next, 150 μl dimethyl sulfoxide (DMSO) was added to dissolve the purple precipitate. The optical absorbance of each sample was recorded at 490 nm using PowerWave XS machine (BioTek, Vermont, USA).

### Tumor cell invasion and migration assay

Tumor cell migration and invasion were assayed using Transwell chambers (8 μm; Corning, Corning, NY, USA) and Matrigel (BD Biosciences, San Jose, CA, USA). In particular, 24 h-transiently transfected HepG2 cells were suspended in serum-free medium at a density of 6 × 10^4^ cells, and 200 μl of the cell suspension were added into the upper chamber, while 500 μl of DMEM containing 10 % FBS was added to the bottom chambers. The plates were cultured for 24 h and the cells on the upper face of the filters were removed with a cotton swab. The migrated or invaded cells on the bottom side of the filters were fixed with 75 % ethanol and stained with 0.5 % crystal violet for 10 min. For the invasion assay, the filters were precoated with 5 μg/ml Matrigel (BD Biosciences).

### Wound scratch assay

HepG2 cells were seeded and transiently transfected with miR-10b or NC for 24 h. After cells reached approximately 100 % confluency, a linear wound was made across the confluent cell layer using 200 μl pipette tips. Cells were then washed twice with phosphate buffered saline (PBS) to remove cell debris and further cultured for 24 h. During the cell culture, wound healing was recorded under an inverted microscope.

### Colony formation assay

HepG2 cells were seeded and transiently transfected with miR-10b or NC for 24 h. 200 cells were then seeded into 60 mm dishes and grown for 2 weeks. Cell colonies were subsequently stained with Giemsa dye. For the soft agar colony formation assay, 5 % agar and complete medium in a 1:9 ratio were mixed at 50 °C, and 0.8 ml was added into each well of a 24-well plate. After the agar was completely solidified at room temperature, 0.8 ml of 0.3 % agar medium containing 40 cells was added to each well covered with solidified agar. After the agar solidified, 0.5 ml DMEM with 10 % FBS was added into each well and the plate was placed in the incubator for 3 weeks during which time the DMEM was replenished twice per week.

### Flow cytometry apoptosis and cell cycle assay

HepG2 cells were seeded and transiently transfected with miR-10b or NC for 24 h. For flow cytometry, cells were collected and stained with the Annexin V-PE/7-AAD apoptosis detection kit (KeyGEN, Nanjing, China) or cell cycle detection kit (KeyGEN) according to the manufacturer’s instructions. Each sample containing 3 × 10^5^ cells was measured by a flow cytometer (Beckman-Coulter, Indianapolis, IN, USA).

### Immunocytochemistry and immunohistochemistry

Immunostaining was carried out using a Streptavidin-labeled peroxidase (S-P) kit (MaiXin Fuzhou, China) according to the manufacturer’s instructions. The primary antibody used was a polyclonal rabbit anti-human CSMD1 antibody at a dilution of 1:200 (Boaosen Ltd. Company, Beijing, China). All of the immunostaining reagents were supplied by MaiXin Biotechnology Corporation Limited (Fuzhou, China). Positive immunostaining with the anti-CSMD1 antibody was assessed when granular brown color was observed in the cytoplasm.

### Tumor cell xenograft assay in nude mice

HepG2 cells at 1 × 10^6^ combined with 200 μl of serum-free DMEM were injected into each nude mouse in the flank region. Following 2 weeks, 50 μl miR-10b mimics or negative control (NC) were injected into the tumor lesion every 3 days (Riobobio, Guangzhou, China). Tumor growth was measured using a caliper every other day, and tumor volume was calculated according to the formula: volume = length × width^2^ × 0.5. Finally, mice were sacrificed and tumor mass was harvested for examination. All studies were performed according to the Chinese Association for the Accreditation of Laboratory Animal Care guidelines for humane treatment of animals and adhered to national and international standards.

### Statistical analysis

All data are expressed as mean ± SD of at least three separate experiments. Differences between groups were analyzed using the paired *t*-test for normal distribution by the F test. All statistical analyses were performed using SPSS 19.0 software (Chicago, IL, USA). A *p* value ≤ 0.05 was considered statistically significant.

## Results

### Overexpression of miR-10b in HCC tissues and hepatoma cell lines

**Fig. 1:**
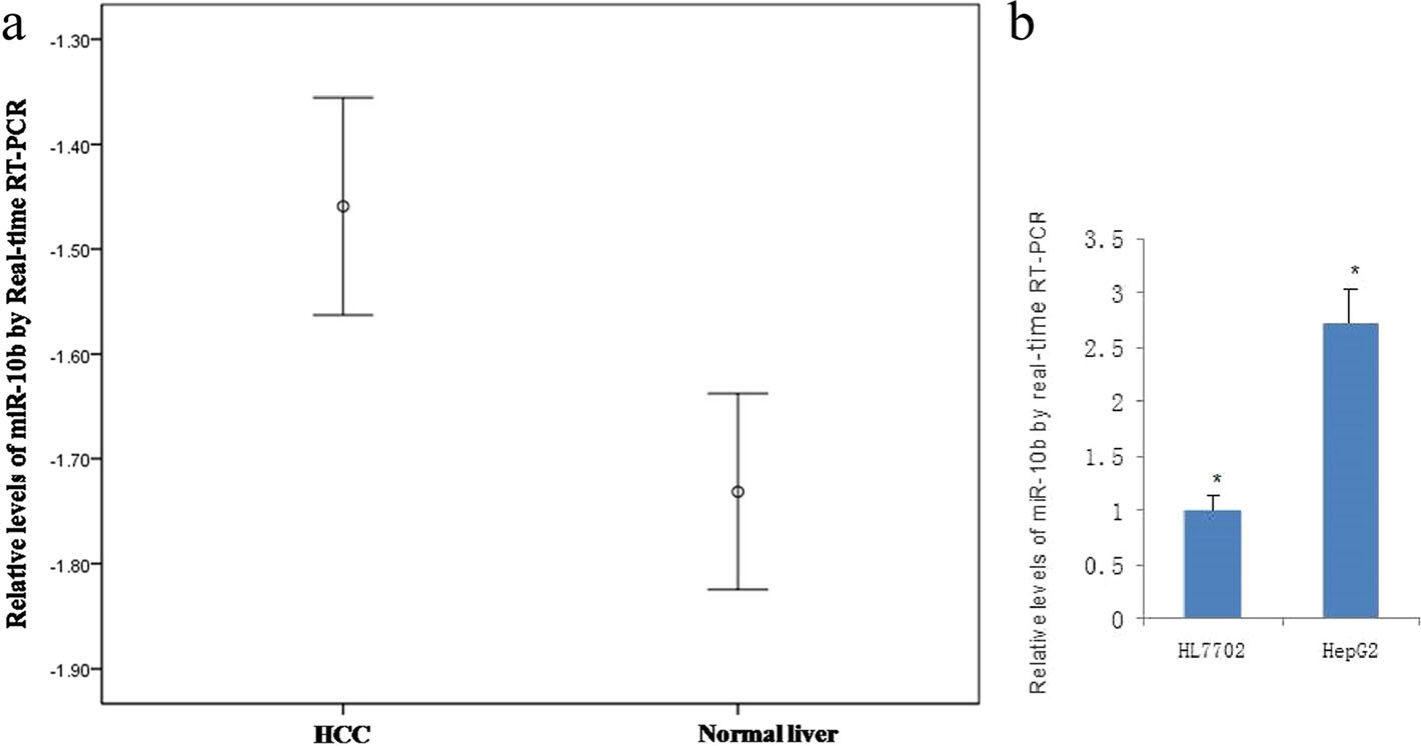
Overexpression of miR-10b in HCC tissues and cells. **a** Relative levels of miR-10b expression in HCC tissues (*n* = 45) and normal liver tissue (*n* = 45) were measured using qRT-PCR. miR-10b levels were higher in HCC samples compared to adjacent nontumor tissues (−1.4590 ± 0.69542 vs. -1.7312 ± 0.62758, *p* < 0.01). **b** The relative levels of miR-10b expression in normal human hepatocytes and HepG2 cells were measured using qRT-PCR. miR-10b expression was nearly 3-fold higher in HepG2 compared to HL-7702 cells

To investigate the role of miR-10b in HCC, we first assessed the expression level of miR-10b in 45 primary HCC and adjacent matched tissues. The results demonstrated that the expression level of miR-10b was higher in HCC samples compared to adjacent non-tumor tissue samples (−1.4590 ± 0.69542 vs. -1.7312 ± 0.62758, *p* < 0.01; Fig. 1a). Similarly, miR-10b expression was nearly 3-fold higher in HepG2 cells compared to HL-7702 cells (Fig. 1b). These data indicate that miR-10b expression is elevated in HCC.

### miR-10b enhances HCC cell viability and colony formation but reduces apoptosis

**Fig. 2:**
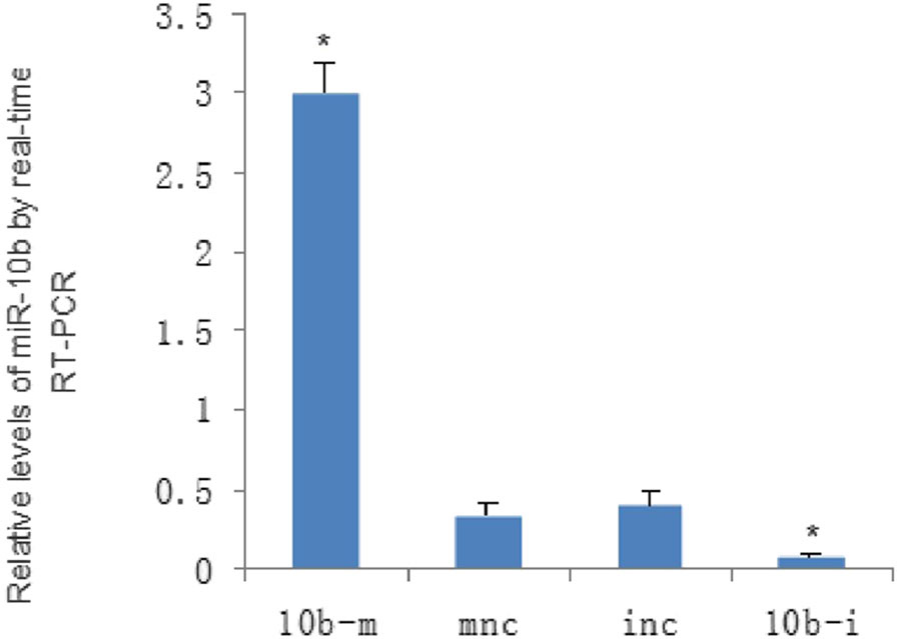
Detection of transient transfection efficiency. We transfected hsa-miR-10b mimics (10b-m), mimics negative control (mnc), hsa-miR-10b inhibitors (10b-i), or inhibitors negative control (inc) into HepG2 cells. Relative levels of miR-10b were measured using qRT-PCR. After transfection of 10b-m, the expression of mir-10b was significantly increased, whereas 10b-i elicited the opposite result

**Fig. 3:**
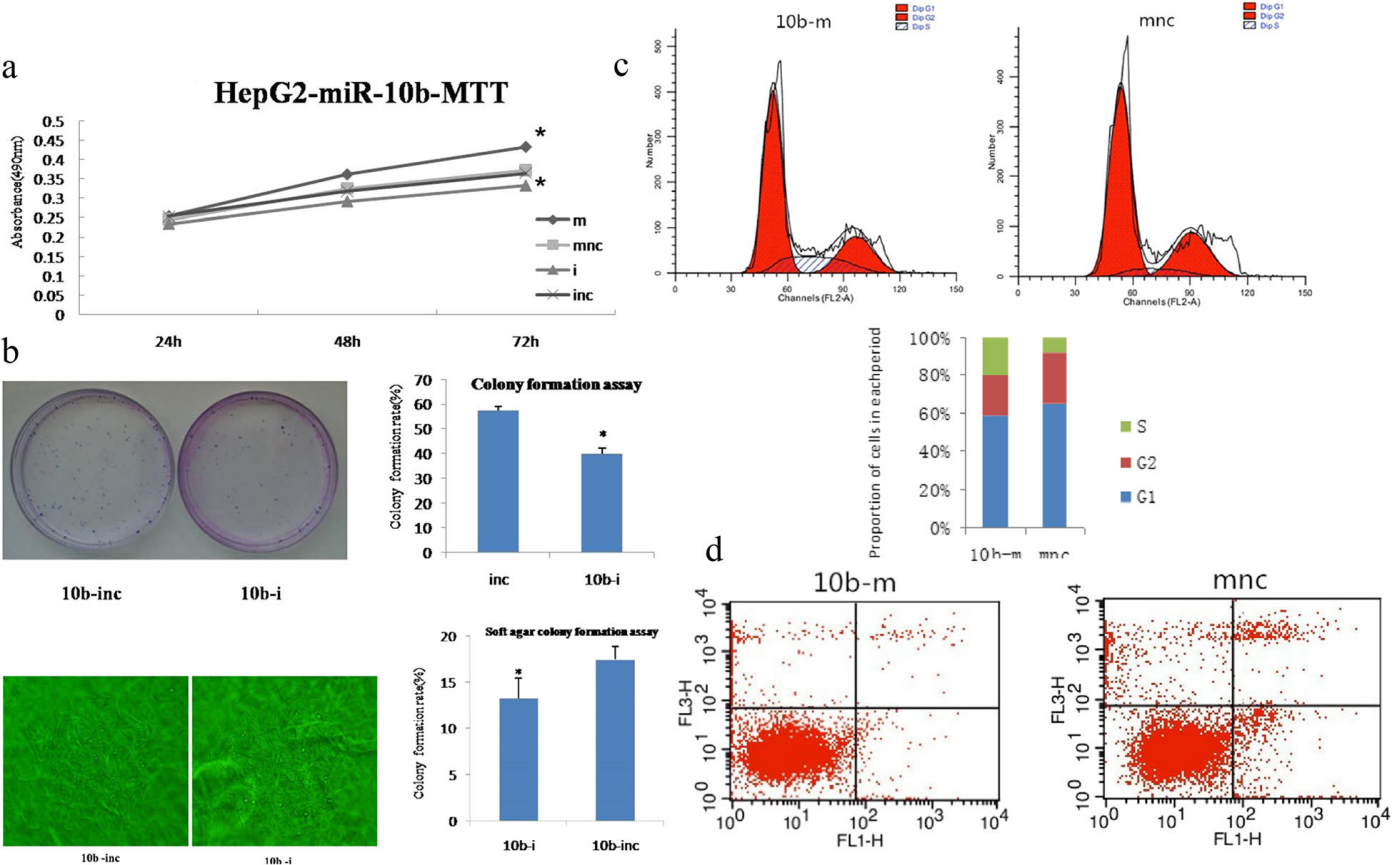
Effects of miR-10b on HepG2 cell viability, colony formation, and apoptosis. HepG2 cells were transfected with hsa-miR-10b mimics (10b-m), mimics negative control (mnc), hsa-miR-10b inhibitors (10b-i), inhibitors negative control (inc). **a** MTT assay. miR-10b mimics increased cell viability after 24–72 h of transfection. In contrast, miR-10b inhibition reduced cell viability. **b** Colony formation and soft agar colony formation assay. miR-10b inhibitors reduced the rate of colony formation by 17.5 and 4.25 %, respectively (*p* < 0.01). **c** Flow cytometry cell cycle assay. 19.3 % of miR-10b mimic-transfected cells were in the S phase of the cell cycle, compared to only 8.02 % of negative control cells (*p* < 0.01). **d** Flow cytometry for apoptosis assessment. miR-10b transfected cells exhibited lower rates of cell death (0.48 % of early apoptotic cells and 0.27 % of late apoptotic cells) compared to their negative control transfected counterparts (1.24 % of early apoptotic cells, 1.24 and 0.91 % of late apoptotic cells; *p* < 0.01)

In HCC cell lines, miR-10b expression was almost 3-fold higher in HepG2 cells compared to HL-7702 cells. To test the oncogenic activity of miR-10b in HCC, we transfected hsa-miR-10b mimics (10b-m), mimics negative control (mnc), hsa-miR-10b inhibitors (10b-i), or inhibitors negative control (inc) into HepG2 cells (Fig. 2). The miR-10b-mediated growth response was evaluated by the MTT assay. As shown in Fig. 3a, miR-10b mimics increased cell viability after 24–72 h transfection. In contrast, miR-10b inhibition reduced cell viability. The effect of miR-10b on cell clonogenic ability was assessed using the colony formation and soft agar colony formation assays. The results showed that the miR-10b inhibitor reduced the rate of colony formation by 17.5 and 4.25 % respectively in colony formation and soft agar colony formation assays (*p* < 0.01, Fig. 3b). Furthermore, flow cytometry was used to analyze cell cycle distribution. 19.3 % of miR-10b mimic-transfected cells were in the S phase of the cell cycle, compared to only 8.02 % of negative control cells (*p* < 0.01, Fig. 3c). As shown in Fig. 3d, miR-10b transfected cells exhibited lower rates of apoptosis (0.48 % of early apoptotic cells and 0.27 % of late apoptotic cells) compared to their negative control transfected counterparts (1.24 % of early apoptotic cells, 1.24 and 0.91 % of late apoptotic cells; *p* < 0.01).

### miR-10b promotes HCC cell migration and invasion

**Fig. 4:**
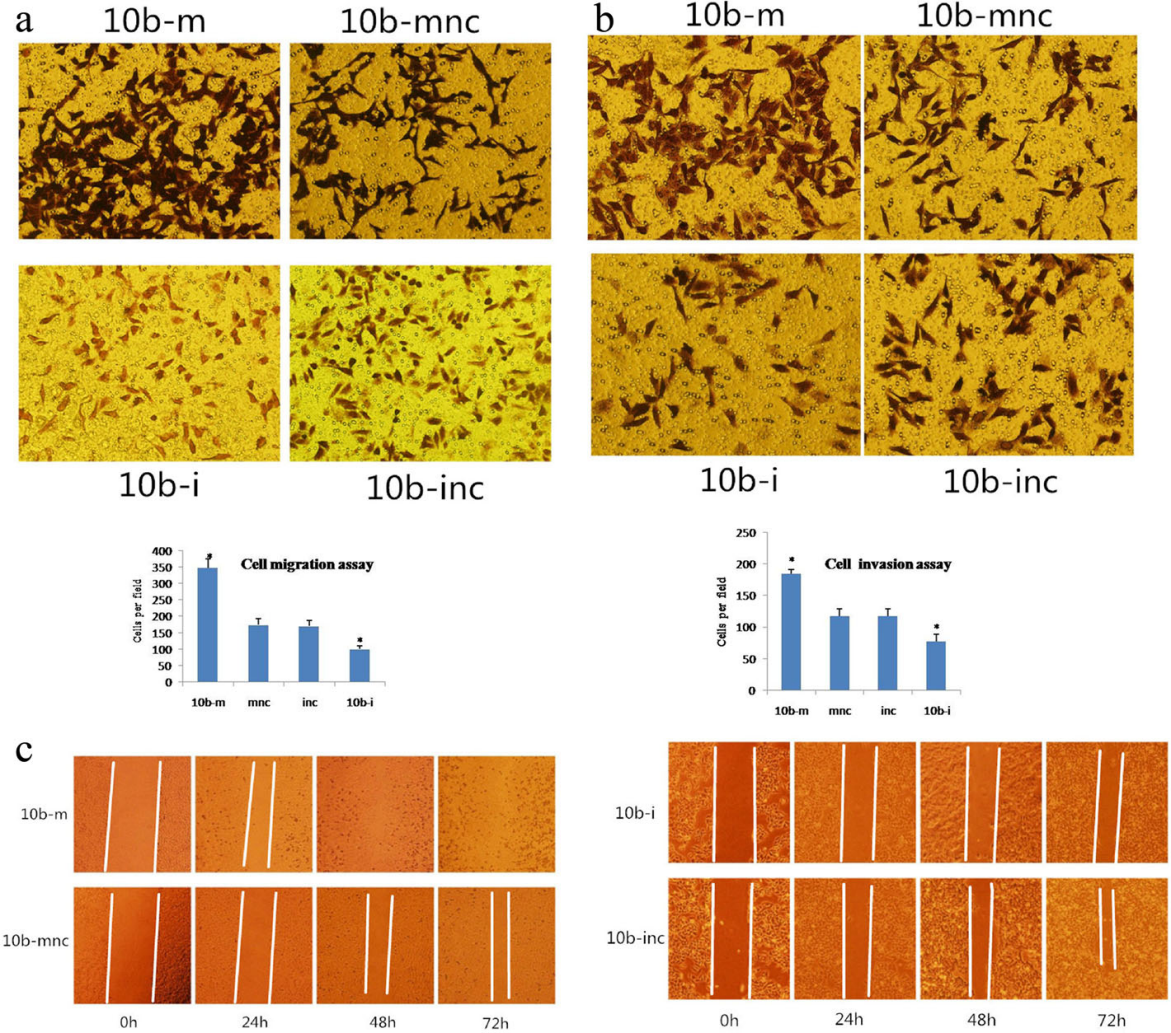
Effects of miR-10b on HCC cell migration and invasion. HepG2 cells were transfected with hsa-miR-10b mimics (10b-m), mimics negative control (mnc), hsa-miR-10b inhibitors (10b-i), or inhibitors negative control (inc). **a** Transwell migration assay. miR-10b mimics led to a 2-fold increase in cell migration, whereas knockdown of miR-10b reduced migration by 40 %. **b** Matrigel invasion assay. miR-10b mimics led to over 50 % increase in cell invasion capacity, whereas knockdown of miR-10b reduced invasion by 40 %. **c** Wound healing assay. miR-10b mimics induced wound healing at 48 h, while the mnc group did not demonstrated wound healing at 72 h. miR-10b inhibitors and inc did not induce wound healing at 72 h, but the inc group demonstrated better wound healing compared to the inhibitors

Next, we assessed the effects of miR-10b on cell migration and invasion in HepG2 cells by overexpressing miR-10b mimics, and using inhibitors of miR-10b as well as their respective negative controls. We found that miR-10b mimics led to a 2-fold increase in cell migration and over 50 % increase in cell invasion capacity. In contrast, tumor cell migration and invasion were reduced by 40 %, respectively (*p* < 0.01, Fig. 4a-b), after knockdown of miR-10b expression. In addition, the wound healing ability of cells overexpressing miR-10b was significantly higher compared to the cells with knockdown of miR-10b expression. miR-10b mimics induced nearly complete wound healing by 48 h, while the mnc group did not demonstrate any healing at 72 h. miR-10b inhibitors or inhibitor negative control (inc) did not induce wound healing at 72 h, but the inc group healed better compared to the inhibitor group. These data indicate that miR-10b overexpression promotes tumor cell viability and migration (*p* < 0.01, Fig. 4c).

### CSMD1 is a gene target of miR-10b in HCC cells

**Fig. 5:**
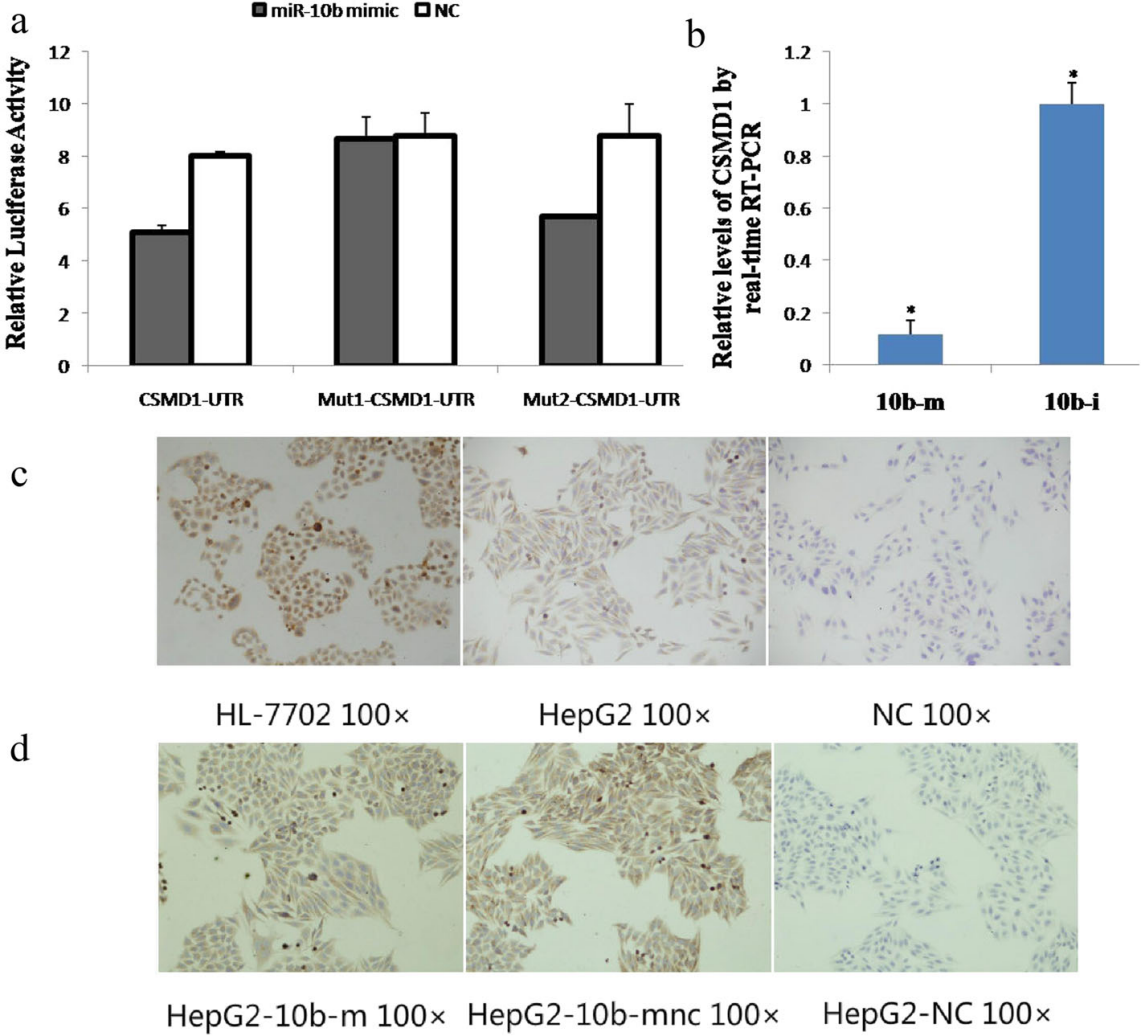
miR-10b binds to CSMD1 3’-UTR and represses CSMD1 expression in HepG2 cells. **a** Luciferase activity assay. HepG2 cells were co-transfected with pMIR/CSMD1 3’-UTR or mutated pMIR mu-CADM1 3’-UTR plus miR-10b mimics or negative control (NC). miR-10b overexpression markedly suppressed luciferase expression in HepG2 cells transfected with pMIR-CSMD1-3’-UTR-WT but not pMIR-CSMD1-3’-UTR-mut1. **b** qRT-PCR. Expression of CSMD1 mRNA after transfection with hsa-miR-10b mimics (10b-m) or hsa-miR-10b inhibitors (10b-i) was measured by qRT-PCR in HepG2. miR-10b overexpression decreased CSMD1 expression; miR-10b knockdown increased CSMD1 expression in cultured cells **c** Immunocytochemistry. CSMD1 expression was low in HepG2 compared to normal liver HL-7702cells. **d** Immunocytochemistry. CSMD1 expression was higher in HepG2 cells transfected with the miR-10b inhibitors, whereas CSMD1 expression was reduced in HepG2 cells transfected with miR-10b mimics

We next performed bioinformatics analyses using online tools of TargetScan, PicTar, miRanda, RNAhybrid, or DIANA-microT, and then identified two miR-10b binding sites in the CSMD1 3’-UTR: 707–713 and 2158–2164. pMIR-CSMD1-3’-UTR-WT contains both the 707–713 and 2158–2164 binding sites. pMIR-CSMD1-3’-UTR-mut1 contains a mutation in the 707–713 (TGTCCCA) site but the 2158–2164 site is normal; pMIR-CSMD1-3’-UTR-mut2 contains a mutation in the 2158–2164 (TGTCCCA) site, but the 707–713 site is normal. We therefore performed a luciferase reporter assay to confirm the binding ability of miR-10b to CSMD1 cDNA and found that miR-10b overexpression markedly suppressed luciferase expression in HepG2 cells transfected with pMIR-CSMD1-3’-UTR-WT but not pMIR-CSMD1-3’-UTR-mut1 (Fig. 5a). These results demonstrate that miR-10b binds to the 707–713 site but not the 2158–2164 site of the CSMD1 3’-UTR.

We next performed qRT-PCR and found that miR-10b overexpression reduced CSMD1 expression, and conversely, knockdown of miR-10b resulted in increased CSMD1 expression in cultured cells (*p* < 0.01, Fig. 5b). Furthermore, CSMD1 expression was low in HepG2 compared to normal liver cells (*p* < 0.01, Fig. 5c). As expected, CSMD1 expression was higher in HepG2 cells transfected with the miR-10b inhibitors, whereas CSMD1 expression was reduced in HepG2 cells transfected with miR-10b mimics (Fig. 5c, *p* < 0.01).

### miR-10b promotes xenograft growth in nude mice

**Fig. 6:**
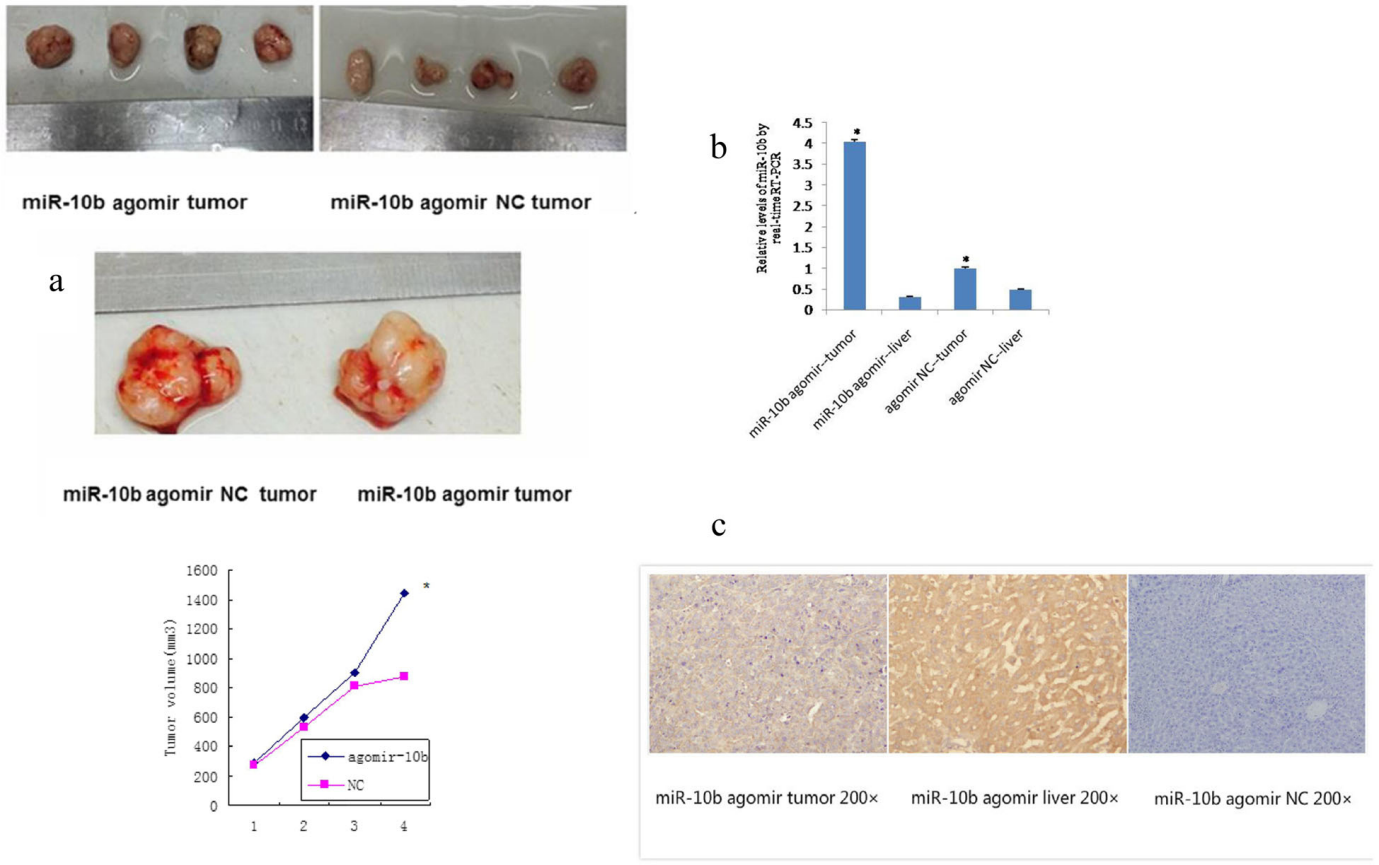
Effects of miR-10b on regulation of tumor cell xenograft growth. miR-10b agomir or agomir negative control (NC) were injected into tumor lesions. **a** miR-10b injection promoted xenograft growth in nude mice. **b** The relative levels of miR-10b expression in xenografts and nude mouse liver tissues were measured by qRT-PCR. The expression level of miR-10b was significantly increased in tumor tissues compared to normal liver tissues. **c** CSMD1 protein expression in xenografts and nude mouse livers was detected by immunohistochemistry. CSMD1 protein expression was decreased in xenografts compared to mouse livers

To further investigate the role of miR-10b in HCC cells, we assessed its oncogenic activity in vivo. Usually, the in vivo half-life of miR-10b mimics is short; thus, we used a miR-10b agomir, a synthetic modified miR-10b analogue with a long in vivo half-life. The data showed that miR-10b injection promoted growth of tumor cell xenografts in nude mice (Fig. 6a). Indeed, qRT-PCR data confirmed miR-10b expression in xenografts (Fig. 6b). Moreover, we found that CSMD1 protein expression was decreased in xenografts compared to mouse liver (Fig. 6c).

### Downregulation of CSMD1 expression promotes HCC cell proliferation, migration, and invasion

**Fig. 7:**
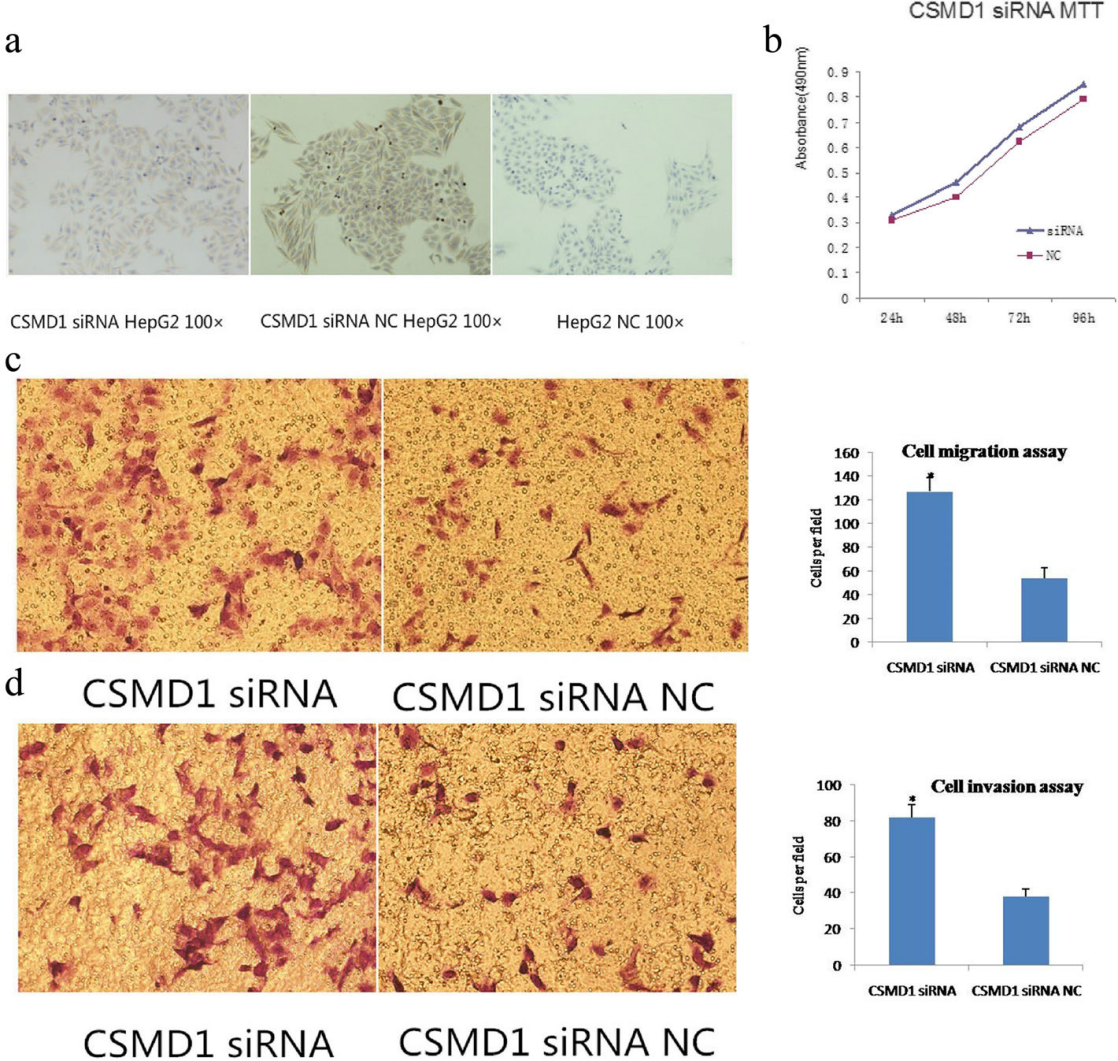
Effects of CSMD1 knockdown on tumor cell viability, invasion, and migration. HepG2 cells were transfected with CSMD1 siRNA or NC **a** CSMD1 protein levels were decreased in HepG2 cells transfected with CSMD1 siRNA. **b** MTT assay. Knockdown of CSMD1 using siRNA promoted proliferation in HepG2 cells. **c** Transwell migration assay. Knockdown of CSMD1 promoted migration in HepG2 cells. **d** Matrigel invasion assay. Knockdown of CSMD1 expression enhanced invasion of HepG2 cells

We then evaluated whether HCC cell growth is regulated by CSMD1 expression. As shown in Fig. 7a, knockdown of CSMD1 using siRNA reduced the expression of CSMD1. Furthermore, knockdown of CSMD1 promoted HepG2 cell proliferation (Fig. 7b). Importantly, HepG2 cell migration and invasion were also induced after CSMD1 knockdown (Fig. 7c, d), suggesting that CSMD1 plays a functional role as a tumor suppressor gene.

## Discussion

Aberrant miRNA expression in various human cancers contributes to cancer development in different phases. miRNAs play an important role in regulating gene expression. miR-10b is located in the HOX gene cluster on chromosome 2, suggesting that it is closely related to tumor invasion and metastasis. Previous studies showed that miR-10b was overexpressed in a variety of human cancers, such as breast cancer, malignant glioma, nasopharyngeal carcinoma, pancreatic cancer, and HCC [10–14]. Consistent with previous reports, our current study showed that miR-10b was overexpressed in HCC tissue samples compared to adjacent non-tumor tissues and in a HCC HepG2 cell line. Up-regulation of miR-10b has been shown to promote invasion and metastasis in breast cancer and esophageal cancer [14, 16]. In our current study, we found that miR-10b also enhanced HepG2 cell migration and invasion in vitro. Further, overexpression of miR-10b inhibited tumor cell apoptosis. Taken together, our data are consistent with previous findings showing that miR-10b promotes HCC metastasis [17], which suggests that miR-10b exerts oncogenic activity in HCC.

miRNAs regulate expression of genes by binding to their target mRNA and marking it for degradation or by blocking protein translation. Generally, individual miRNAs regulate multiple target genes, while several miRNAs can also regulate a single gene. Previous studies have shed light on the relationship between miR-10b and cancer. Some studies have revealed that miR-10b promotes metastasis of glioma cells through regulation of HOXD10, Bim, TFAP2C, P16, and P21 [13, 18]. Other studies have shown that miR-10b induces invasion of breast cancer through targeting E-cadherin, and Syndecan-1 [19, 20]. In HCC, miR-10b induces cell invasion by modulating RhoC, uPAR, MMP-2, and MMP-9 via HOXD10 [21]. Furthermore, CSMD1 participates in endothelial-to-mesenchymal transition (EndoMT), is a direct target of miR-10b [10, 22]. In our study, we further explored the molecular mechanism between miR-10b and CSMD1. Bioinformatics analyses revealed that there are two binding sites for miR-10b in the CSMD1 3’ UTR: 707–713 and 2158–2164. Luciferase reporter assays showed that miR-10b could bind to the 707–713 site but not the 2158–2164 site.

CSMD1 is localized at chromosome 8p23.2 [23] and allelic imbalance and chromosomal aberrations of chromosome 8 are associated with development of many cancers. Studies have shown that CSMD1 loss is a common phenomenon in breast, lung, prostate, and head and neck cancers [24]. Similarly, Midorikawa et al., observed homozygous deletion and loss of heterozygosity of 8p23.2 in HCC [25, 26]. Generally, homozygous deletion is often related to tumor suppressor genes in cancer. These findings suggest that CSMD1 is a putative tumor suppressor gene. Surprisingly, homologous structures, namely the CUB and SUSHI domains, are shared between CSMD1 and other proteins that play important roles in cancer progression, such as SEZ6L and DMBT1 [27, 28]. Last but not least, low CSMD1 expression is closely related to high tumor grade in a variety of cancers. Also, studies have shown that deletion of CSMD1 is associated with poor prognosis in head and neck squamous cell carcinoma and prostate cancer [24, 29]. Taken together, mounting evidence indicates that CSMD1 functions as a tumor suppressor gene.

In our previous study, we found that CSMD1 expression is lower in HCC, which is consistent with the results in breast cancer and melanoma [29, 30]. Compared to normal liver cells, CSMD1 expression was downregulated in HepG2 cells. We used siRNA-mediated silencing of CSMD1 in order to further clarify the role of CSMD1 in tumor cell proliferation and invasion. Our findings are consistent with previous observations regarding the effects of miR-10b overexpression. Research has found that CSMD1 can activate the Smad pathway to increase antitumor activity [29]. Tumor growth factor-β (TGF-β) activates this pathway in two ways via Smad2/3 and Smad 1/5/8. Also, Morris et. al., reported that TGF-β can promote hepatocarcinogenesis by inducing p53-deficiency [31]. Some studies have demonstrated that the TGF-β/Smad pathway can cause cell cycle arrest through high expression p15, p21 and p27. These pathways may be associated with the tumor suppressor role of CSMD1.

## Conclusion

In conclusion, we analyzed miR-10b expression in HCC tissue samples and investigated its effects on cells and found that overexpression of miR-10b enhanced HCC cell viability, migration, and invasion. Our study demonstrates that CSMD1 is indeed a direct target of miR-10b in HCC, and miR-10b can mediate an oncogenic effect in HCC by targeting CSMD1. These findings provide important information toward the goal of developing miR-10b and CSMD1 as promising candidates for effective HCC therapeutic strategies.

## Abbreviations

CSMD1: CUB and Sushi multiple domains 1
HCC: Hepatocellular carcinoma
MTT: 3-(4,5-dimethylthi azol-2-yl)-2,5-diphenyltetrazolium bromide
TGF-β: Tumor growth factor-β

## References

[1] Jemal A, Bray F, Center MM, Ferlay J, Ward E, Forman D (2011). Global cancer statistics. CA Cancer J Clin.

[2] El-Serag HB (2007). Epidemiology of hepatocellular carcinoma in USA. Hepatol Res.

[3] Parkin DM, Bray F, Ferlay J, Pisani P (2005). Global cancer statistics, 2002. CA Cancer J Clin.

[4] Pascual S, Herrera I, Irurzun J (2016). New advances in hepatocellular carcinoma. World J Hepatol.

[5] Chan SH, Wu CW, Li AF, Chi CW, Lin WC (2008). miR-21 microRNA expression in human gastric carcinomas and its clinical association. Anticancer Res.

[6] Pillai RS, Bhattacharyya SN, Artus CG, Zoller T, Cougot N, Basyuk K (2005). Inhibition of translational initiation by Let-7 MicroRNA in human cells. Science.

[7] Esquela-Kerscher A, Slack FJ (2006). Oncomirs-microRNAs with a role in cancer. Nat Rev Cancer.

[8] Nelson KM, Weiss G (2008). MicroRNAs and cancer: past, present, and potential future. Mol Cancer Ther.

[9] Winter J, Diederichs S (2011). MicroRNA biogenesis and cancer. Methods Mol Biol.

[10] Ladeiro Y, Couchy G, Balabaud C, Bioulac-Sage P, Pelletier L, Rebouissou S (2008). MicroRNA profiling in hepatocellular tumors is associated with clinical features and oncogene/tumor suppressor gene mutations. Hepatology.

[11] Li G, Wu Z, Peng Y, Liu X, Lu J, Wang L (2010). MicroRNA-10b induced by Epstein-Barr virus-encoded latent membrane protein-1 promotes the metastasis of human nasopharyngeal carcinoma cells. Cancer Lett.

[12] Nakata K, Ohuchida K, Mizumoto K, Kayashima T, Ikenaga N, Sakai H (2011). MicroRNA-10b is overexpressed in pancreatic cancer, promotes its invasiveness, and correlates with a poor prognosis. Surgery.

[13] Sasayama T, Nishihara M, Kondoh T, Hosoda K, Kohmura E (2009). MicroRNA-10b is overexpressed in malignant glioma and associated with tumor invasive factors, uPAR and RhoC. Int J Cancer.

[14] Ma L, Teruya-Feldstein J, Weinberg RA (2007). Tumour invasion and metastasis initiated by microRNA-10b in breast cancer. Nature.

[15] Y, Y. Mechanism of miRNA-210 and its Target Gene SIN3A in Tumor Invasion and Metastasis of Hepatocellular Carcinoma. The second military medical university; 2012.

[16] Tian Y, Luo A, Cai Y, Su Q, Ding F, Chen H (2010). MicroRNA-10b promotes migration and invasion through KLF4 in human esophageal cancer cell lines. J Biol Chem.

[17] Li QJ, Zhou L, Yang F, Wang GX, Zheng H, Wang DS (2012). MicroRNA-10b promotes migration and invasion through CADM1 in human hepatocellular carcinoma cells. Tumour Biol.

[18] Gabriely G, Yi M, Narayan RS, Niers JM, Wurdinger T, Imitola J (2011). Human glioma growth is controlled by microRNA-10b. Cancer Res.

[19] Liu Y, Zhao J, Zhang PY, Zhang Y, Sun SY, Yu SY, et al. MicroRNA-10b targets E-cadherin and modulates breast cancer metastasis. Med Sci Monit. 2012;18:BR299–308. 10.12659/MSM.883262 PMC3560697 22847191

[20] Ibrahim SA, Yip GW, Stock C, Pan JW, Neubauer C, Poeter M (2012). Targeting of syndecan-1 by micorRNA miR-10b promotes breast cancer cell motility and invasiveness via a Rho-GTPase- and E-cadherin-dependent mechanism. Int J Cancer.

[21] Liao CG, Kong LM, Zhou P, Yang XL, Huang JG, Zhang HL, et al. miR-10b is overexpressed in hepatocellular carcinoma cell proliferation, migration and invasion through RhoC, uPAR and MMPs. J Transl Med. 2014;12:234. 10.1186/s12967-014-0234-x PMC4192292 25236186

[22] Sakurai-Yageta M, Masuda M, Tsuboi Y, Ito A, Murakami Y (2009). Tumor suppressor CADM is involved in epithelial cell structure. Biochem Biophys Res Commun.

[23] Sun PC, Uppaluri R, Schmidt AP, Pashia ME, Quant EC, Sunwoo JB (2001). Transcript map of the 8p23 putative tumor suppressor region. Genomics.

[24] Ma C, Quesnelle KM, Sparano A, Rao S, Park MS, Cohen MA (2009). Characterization CSMD1 in a large set of primary lung, head and neck, breast and skin cancer tissues. Cancer Biol Ther.

[25] Midorikawa Y, Yamamoto S, Ishikawa S, Kamimura N, Igarashi H, Sugimura H (2006). Molecular karyotyping of human hepatocellular carcinoma using single-nucleotide polymorphism arrays. Oncogene.

[26] Midorikawa Y, Yamamoto S, Tsuji S, Kamimura N, Ishikawa S, Igarashi H (2009). Allelic imbalances and homozygous deletion on 8p23.2 for stepwise progression of hepatocarcinogenesis. Hepatology.

[27] Nishioka M, Kohno T, Takahashi M, Niki T, Yamada T, Sone S,e tal. Identification of a 428-kb homozygously deleted region disrupting the SEZ6L gene at 22q12.1 in a lung cancer cell line. Oncogene. 2000; 19:6251–60. 10.1038/sj.onc.1204031 11175339

[28] Mollenhauer J, Helmke B, Medina D, Bergmann G, Gassler N, Muller H (2004). Carcinogen inducibility in vivo and down-regulation of DMBT1 during breast carcinogenesis. Genes Chromosomes Cancer.

[29] Kamal M, Shaaban AM, Zhang L, Walker C, Gray S, Thakker N (2010). Loss of CSMD1 expression is associated with high tumour grade and poor survival in invasive ductal breast carcinoma. Breast Cancer Res Treat.

[30] Tang MR, Wang YX, Guo S, Han SY, Wang D (2012). CSMD1 exhibits antitumor activity in A375 melanoma cells through activation of the Smad pathway. Apoptosis.

[31] Morris SM, Baek JY, Koszarek A, Knoblaugh SE, Knoblaugh SE, Grady WM (2012). Transforming growth factor-beta signaling promotes hepatocarcinogenesis induced by p53 loss. Hepatology.

